# Closure of small and medium size umbilical hernias with the Proceed Ventral Patch in obese patients: a single center experience

**DOI:** 10.1186/2193-1801-3-686

**Published:** 2014-11-24

**Authors:** Dirk Wassenberg, Nikolaos Zarmpis, Nora Seip, Peter C Ambe

**Affiliations:** Helios Klinikum Wuppertal, Department of Surgery II, Witten/Herdecke University, Heusner Str. 40, 42283 Wuppertal, Germany; Department of general, visceral and thoracic surgery, St. Remigius Hospital Opladen, An St. Remigius 26, 51379 Leverkusen, Germany

**Keywords:** Umbilical hernia, Obesity, Proceed Ventral Patch, Hernia recurrence, Ventral hernia

## Abstract

Obesity is a risk factor for the development of umbilical hernia. Open hernia closure could be challenging in obese patients leading to high rates of recurrence. The aim of this study was to investigate the effectiveness and safety of hernia patches in the management of obese patients with umbilical hernias. All the patients included in this study were managed in the department of surgery of a primary care hospital in Germany. The data of patients undergoing umbilical hernia repair within a two-year period was retrospectively reviewed. Patients managed with the PVP were included for analysis. 24 obese patients were analyzed. Small and medium size patches were used in 15 and 9 patients respectively. The median duration of surgery was 40 min and the median length of hospital stay was 4d. The mean length of follow-up was 12 ± 9 months (range: 6–30 months). The rate of recurrence was 4.1% and the rate of complication was 8.3%. Obese patients presenting with small and medium size umbilical hernias could be safely and effectively managed with prosthetic patches like the Proceed Ventral Patch. However, the limited overlap zone following hernia closure with such a patch can be an issue.

## Background

Obesity predisposes to a number of medical conditions. Current literature suggests obesity to be a risk factor for the development of umbilical hernias (UH) (Shenkman et al. 1993; Smith et al. 2004). Furthermore, obesity has been shown to influence not only surgical outcome but also the rate of recurrence in patients undergoing ventral hernia repair (Regnard et al. 1988; Sugerman et al. 1996; Sauerland et al. 2004).

Hernia closure with a mesh has been shown to be superior to suture repair with regard to recurrence (Arroyo et al. 2001; Luijendijk et al. 2000). Minimal invasive mesh associated techniques have been shown to reduce postoperative complications in obese patients undergoing ventral hernia repair (Birgisson et al. 2001; Colon et al. 2013; Eid et al. 2013). Over the last decade, prosthetic patches have been employed in the closure of UH (Berrevoet et al. 2011; Iversen et al. 2010; Tollens et al. 2010). Current data supports hernia patches to be safe and effective in the management of small and medium size ventral hernias including UH (Iversen et al. 2010; Ambe et al. 2013; Martin et al. 2008). However, no data exists on the use of hernia patches in obese patients. The aim of this series therefore, was to investigate the safety and effectiveness of multilayered polytetrafluoroethylene (ePTFE) free, partially absorbable and self-expanding hernia patches in the closure of small and medium size umbilical hernias in obese patients.

## Methods and procedures

Following the approval of the hospital’s ethic committee, a retrospective analysis of prospectively collected data of patients undergoing umbilical hernia repair for symptomatic, i.e. painful UH, from January 2012 to December 2013 in the department of surgery of a primary care center in Germany was performed. Baseline characteristics including age, sex, body mass index (BMI) and medical comorbidities as defined by the American Society of Anesthesiologists (ASA) were recorded for each patient. Only obese patients (as defined by the World Health Organisation, WHO) managed with multilayered polytetrafluoroethylene (ePTFE) free, partially absorbable and self-expanding hernia patche*s* were included for analysis.

Surgery was performed either by an experienced member of the surgical team (attending or fellow) or by a surgical resident under direct supervision. All surgeries were performed in general anesthesia as inpatient procedures and a single shot antibiotic was administered in all cases. Surgery began with an infra-umbilical semilunar incision, which was carried down to the fascia to expose and measure the diameter of the hernia defect. Small hernia sacs were reduced without resection while larger sacs were resected and the peritoneum closed using absorbable sutures. The patch was then placed into the preperitoneal space tension free, after digital adhesiolysis. The straps were then pulled to ensure its correct positioning against the abdominal wall. The straps were then sutured to the fascia with non-absorbable sutures, after which the overlaps were resected as previously described (Ambe et al. 2013). In order to achieve a tension free repair, the edges of the fascial defect were not approximated. The subcutis was adapted using absorbable sutures followed by skin closure.

The hernia defect was classified using the classification proposed by the European Hernia Society, i.e. small hernias had diameters <2 cm, medium size hernias had diameters between 2–4 cm and large hernias had diameters >4 cm (Muysoms et al. 2009). The patch size was chosen to ensure maximum coverage of the hernia defect. Thus defects with diameters of 1–2 cm were managed with small patches (4.3 cm in diameter) while those with diameters between 2–4 cm were managed with medium patches (6.4 cm in diameeter). Defects >4 cm in diameter were managed with meshes placed at the retromuscular position using the sublay technique while those with diameters <1 cm were closed using non-absorbable sutures. These cases were excluded from the study.

Perioperative data including the duration of surgery (time from incision to suture) and the size of the implanted patch were retrieved from surgical documentation sheets and surgeon’s notes. Information on postoperative complications and the length of postoperative hospital stay were taken from the final discharge notes. Generally, follow-up after hospital discharge was with the referring physician. Unclear and complicated cases were referred back to our department where follow-up was continued as needed. Physical examination and ultrasound sonography were performed in every patient by an experienced member of the surgical team. All patients included in this series were last followed up in our outpatient office in May 2014.

The data collected was analyzed using the Statistical Package for Social Science (SPSS^®^), IBM, version 22. The study population was statistically described using absolute case numbers and percentages, while central tendencies were described using means, medians, standard diviations and interquartile ranges.

## Results

**Figure 1:**
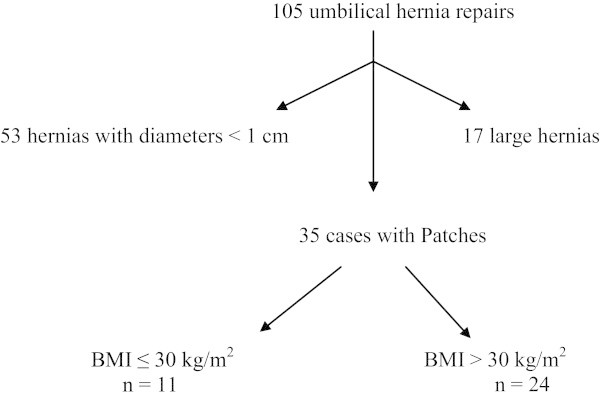
**Distribution of the study population.** The proceed ventral patch was employed in 35 cases. 24 patients met the WHO definition of obesity.

**Figure 2:**
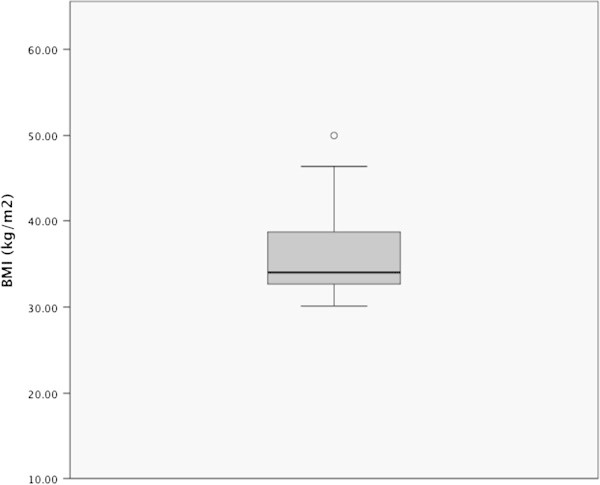
**Distribution of BMI.** The median BMI of the study population was 34 kg/m^2^.

Within the period of investigation 105 patients with umbilical hernias were surgically managed in our department. Hernia patches were employed in 35 cases, 24 of these cases met the WHO definition for obesity (BMI >30 kg/m^2^) and constituted the population examined, Figure 1. The demographic characteristics of the study population are summarized in Table 1. The distribution of the BMI in the study population is presented in Figure 2, while the perioperative features are summarized in Table 2.

**Table 1:** **Demographic characteristics**

Parameters	Number of cases
Gender (female/male)	6/18
Median age (yrs)	54
Interquartile range (yrs)	8
Median BMI (kg/m^2^)	34
Interquartile range (kg/m^2^)	7.8
ASA 1	3
2	18
3	3

Summary of the baseline features of the study population. BMI: body mass index, yrs: years, ASA: American Society of Anesthesiologists.

**Table 2:** **Perioperative characteristics**

Characteristics	Results
Patch size:	
- small (4.3 × 4.3 cm)	15
- medium (6.4 × 6.4 cm)	9
Surgical expertise:	
- attending/fellow	8
- resident	16
Median length of surgery (min)	40
Interquartile range (min)	18
Median length of stay (d)	4
Interquartile range (d)	2

Summary of the perioperative features of the study population.

Min: minutes, d: days.

Hernia defects with diameters between 1–2 cm were managed in 15 cases (62.5%) with small patches (4.3 cm in diameter). Medium defects, i.e. defects with diameters of 2–4 cm were seen in 9 cases (37.5%) and were closed using medium size patches (6.4 cm in diameter).

Over 60% (16 cases) of the procedures were performed by surgical residents under supervision. The median duration of surgery was 40 minutes while the median length of postoperative hospital stay was 4 days, Table 2.

All 24 cases included in this study were last seen in our outpatient office in May 2014. The mean length of follow-up was 12 ± 9 months (range: 6 – 30 months). A follow - up period of up to 12 months was recorded in five cases. A Follow – up period of 12 to 24 months was recorded in 10 cases, while nine cases were followed up for >24 months (26 –30 months).

Two complications (8.3%) including one case of postoperative hematoma and one case of wound infection were recorded in this series. Recurrence was recorded in one patient (4.1%). There was no mortality in this series.

## Discussion

Surgical management of umbilical hernia in obese patients can be challenging. Fascial closure following open procedures could be difficult resulting to an insufficient tissue approximation, thereby predisposing to recurrence (Israelsson & Jonsson 1997). Thus obesity is a risk factor for the development of incisional hernia (Mendoza et al. 1996; Pikarsky et al. 2002). However, increasing expertise in minimal invasive management of ventral hernias has led to a great decrease in the rate of complication following ventral hernia closure (Heniford et al. 2003; Lee et al. 2013). Over the last years, many studies on the use of prosthetic hernia patches for the management of small and medium size umbilical and ventral hernia have been published (Iversen et al. 2010; Tollens et al. 2010; Ambe et al. 2013; Martin et al. 2008). However, none of the available series focused on obese patients.

We aimed at investigating whether or not this technique could be a safe and effective option for the management of obese patients with small and medium size umbilical hernias. Prospectively collected data of patients undergoing UH repair for symptomatic UH, i.e. painful UH, was retrospectively analyzed. Only patients meeting the WHO definition of obesity, i.e. patients with BMI >30 kg/m^2^ undergoing umbilical hernia repair with multilayered polytetrafluoroethylene (ePTFE) free, partially absorbable and self-expanding hernia patches were included for analysis.

Twenty four cases, 15 with small size and nine with medium size defects were included for analysis.

The size of the hernia patch was chosen to achieve maximum defect coverage. Thus small hernias with diameters of 1–2 cm were closed with small size patches (4.3 cm in diameter), while defects with diameters between 2–4 cm were closed with medium size patches (6.4 cm in diameter). Although such patches are generally placed intraperitoneally, all patches in this series were placed in the preperitoneal position with the hope of preventing peritoneal adhesions and other patch- related bowel complications.

The median BMI in this series was 34 kg/m^2^. The median duration of surgery was 40 min while the median length of postoperative hospital stay was 4 days. The median length of hospital stay in this series is rather long compared to the complexity of the procedure, especially since the procedure can be performed on an outpatient basis. This trend must be blamed on the local health insurance policy in Germany.

Complications were recorded in 8.3% in this series. The two complications recorded included a case of postoperative hematoma which was managed conservatively and a case of wound infection. Infection was limited to the subcutis. This case was managed by bedside wound opening and open wound dressing. Complication rates between 2.2% to 11.8% have been reported by Martin et al. and Iversen et al. for intraperitoneally placed patches (Iversen et al. 2010; Martin et al. 2008). Furthermore, the 8.3% incidence of postoperative complication reported in this series is comparable with the 8% incicence for laparoscopic repair and is way below the 21% rate for open repair reported by McGreevy et al. (McGreevy et al. 2003).

The rate of recurrence was 4.1% in this series. Recurrence occurred within one year after hernia closure with a small patch. Recurrence was evident via umbilical protusion and was confirmed both clinically and following ultrasound sonograph. The patch was removed and the hernia was closed with a mesh in the sublay technique. In this case, the patch was probably too small to prevent recurrence since no significant shrinkage was noted after patch removal.

The rate of recurrence in this series was comparable to the 3.5% rate reported by Sanchez et al. (Sanchez et al. 2004) for laparoscopic repair and the 3.6% rate reported by Berrevoet et al. for retromuscular repair (Berrevoet et al. 2011).

Although the patch sizes used in this series are in accordance with current literature, the narrow overlap is an issue.

Closing a small size hernia of about 2 cm in diameter with a small patch (4.3 cm diameter) permits a maximum overlap of 1.15 cm. Similarly, a maximum overlap of 1.2 cm is to be expected after closing a 4 cm defect using a medium patch (6.4 cm diameter). It is therefore questionable if this limited overlap provides sufficient closure to prevent recurrence. In fact, the recurrence seen in this series must be blamed on a rather insufficient overlap. Using a larger patch, i.e. closing small defects with medium patches, could increase the overlapping zone. Digital dissection of the preperitoneal space was possible via the hernia defect. However, introducing a larger patch via a small defect was challenging. Surgically increasing the size of the defect would have enabled the placement of a larger patch. This however, would have been at the expense of the overlapping zone and was therefore not performed. Unfortunately, data on long-term outcome of patients managed with this technique with regard to recurrence is lacking.

This patch associated closure technique utilizes a small skin incision. The minimal invasive nature of this technique is not limited to the size of the incision. In fact, extensive dissection, which may increase the risk of postoperative complication is usually not necessary. Another advantage of this closure method is the relatively short duration of surgery.

Taken together, our results suggest that hernia patches in the preperitoneal position could be an option in the management of obese patients with small and medium size umbilical hernias. The rates of complication and recurrence are comparable with existing data. The small size of the incision, the short duration of surgery and the fact that the procedure can be performed on an outpatient basis comprise the advantages of this technique.

### Limitations

The results presented in this series are mainly limited by the small size of the study population and relatively short follow-up period. Thus the trends reported in this series must be validated in studies with larger case numbers and longer follow-up periods.

## Conclusion

Hernia patches could be a safe and effective option for the management of small and medium size umbilical hernias in obese patients. The advantages of this technique include a small incision, limited tissue trauma and short duration of surgery. The rates of morbity and recurrence are comparable with current data. However, the limited overlap zone associated with this technique warrents further investigation.

### Consent

A written informed consent was obtained from each patient included in this series.

## References

[CR1] Ambe P, Meyer A, Kohler L (2013). Repair of small and medium size ventral hernias with a Proceed Ventral Patch: a single center retrospective analysis. Surg Today.

[CR2] Arroyo A, Garcia P, Perez F, Andreu J, Candela F, Calpena R (2001). Randomized clinical trial comparing suture and mesh repair of umbilical hernia in adults. Br J Surg.

[CR3] Berrevoet F, D’Hont F, Rogiers X, Troisi R, Hemptinne B (2011). Open intraperitoneal versus retromuscular mesh repair for umbilical hernias less than 3 cm diameter. Am J Surg.

[CR4] Birgisson G, Park AE, Mastrangelo MJ, Witzke DB, Chu UB (2001). Obesity and laparoscopic repair of ventral hernias. Surg Endosc.

[CR5] Colon MJ, Kitamura R, Telem DA, Nguyen S, Divino CM (2013). Laparoscopic umbilical hernia repair is the preferred approach in obese patients. Am J Surg.

[CR6] Eid GM, Wikiel KJ, Entabi F, Saleem M (2013). Ventral hernias in morbidly obese patients: a suggested algorithm for operative repair. Obes Surg.

[CR7] Heniford BT, Park A, Ramshaw BJ, Voeller G (2003). Laparoscopic repair of ventral hernias: nine years’ experience with 850 consecutive hernias. Ann Surg.

[CR8] Israelsson LA, Jonsson T (1997). Overweight and healing of midline incisions: the importance of suture technique. Eur J Surg.

[CR9] Iversen E, Lykke A, Hensler M, Jorgensen LN (2010). Abdominal wall hernia repair with a composite ePTFE/polypropylene mesh: clinical outcome and quality of life in 152 patients. Hernia.

[CR10] Lee J, Mabardy A, Kermani R, Lopez M, Pecquex N, McCluney A (2013). Laparoscopic vs open ventral hernia repair in the era of obesity. JAMA Surg.

[CR11] Luijendijk RW, Hop WC, van den Tol MP, de Lange DC, Braaksma MM, IJzermans JN, Boelhouwer RU, de Vries BC, Salu MK, Wereldsma JC, Bruijninckx CM, Jeekel J (2000). A comparison of suture repair with mesh repair for incisional hernia. N Eng J Med.

[CR12] Martin DF, Williams RF, Mulrooney T, Voeller GR (2008). Ventralex mesh in umbilical/epigastric hernia repairs: clinical outcomes and complications. Hernia.

[CR13] McGreevy JM, Goodney PP, Birkmeyer CM, Finlayson SR, Laycock WS, Birkmeyer JD (2003). A prospective study comparing the complication rates between laparoscopic and open ventral hernia repairs. Surg Endosc.

[CR14] Mendoza D, Newman RC, Albala D, Cohen MS, Tewari A, Lingeman J, Wong M, Kavoussi L, Adams J, Moore R, Winfield H, Glascock JM, Das S, Munch L, Grasso M, Dickinson M, Clayman R, Nakada S, McDougall EM, Wolf IS, Hulbert J, Leveillee RJ, Houshair A, Carson C (1996). Laparoscopic complications in markedly obese urologic patients (a multi-institutional review). Urology.

[CR15] Muysoms FE, Miserez M, Berrevoet F, Campanelli G, Champault GG, Chelala E, Dietz UA, Eker HH, El Nakadi I, Hauters P, Hidalgo Pascual M, Hoeferlin A, Klinge U, Montgomery A, Simmermacher RK, Simons MP, Smietański M, Sommeling C, Tollens T, Vierendeels T, Kingsnorth A (2009). Classification of primary and incisional abdominal wall hernias. Hernia.

[CR16] Pikarsky AJ, Saida Y, Yamaguchi T, Martinez S, Chen W, Weiss EG, Nogueras JJ, Wexner SD (2002). Is obesity a high-risk factor for laparoscopic colorectal surgery?. Surg Endosc.

[CR17] Regnard JF, Hay JM, Rea S, Fingerhut A, Flamant Y, Maillard JN (1988). Ventral incisional hernias: incidence, date of recurrence, localization and risk factors. Ital J Surg Sci.

[CR18] Sanchez LJ, Bencini L, Moretti R (2004). Recurrences after laparoscopic ventral hernia repair: results and critical review. Hernia.

[CR19] Sauerland S, Korenkov M, Kleinen T, Arndt M, Paul A (2004). Obesity is a risk factor for recurrence after incisional hernia repair. Hernia.

[CR20] Shenkman Z, Shir Y, Brodsky JB (1993). Perioperative management of the obese patient. Br J Anaesth.

[CR21] Smith RL, Bohl JK, McElearney ST, Friel CM, Barclay MM, Sawyer RG, Foley EF (2004). Wound infection after elective colorectal resection. Ann Surg.

[CR22] Sugerman HJ, Kellum JM, DeMaria EJ, Reines HD (1996). Conversion of failed or complicated vertical banded gastroplasty to gastric bypass in morbid obesity. Am J Surg.

[CR23] Tollens T, Struyve D, Aelvoet C, Vanrijkel JP (2010). Introducing the Proceed Ventral Patch as a new device in surgical management of umbilical and small ventral hernias: preliminary results. Surg Technol Int.

